# The psychometric properties of the 10-item Kessler Psychological Distress Scale (K10) in Canadian military personnel

**DOI:** 10.1371/journal.pone.0196562

**Published:** 2018-04-26

**Authors:** Hugues Sampasa-Kanyinga, Mark A. Zamorski, Ian Colman

**Affiliations:** 1 School of Epidemiology and Public Health, University of Ottawa, Ottawa, Ontario, Canada; 2 Directorate of Mental Health, Canadian Forces Health Services Group, Ottawa, Ontario, Canada; 3 Department of Family Medicine, University of Ottawa, Ottawa, Ontario, Canada; Universidad Miguel Hernandez de Elche, SPAIN

## Abstract

The psychometric properties of the ten-item Kessler Psychological Distress scale (K10) have been extensively explored in civilian populations. However, documentation of its psychometric properties in military populations is limited, and there is no universally accepted cut-off score on the K10 to distinguish clinical vs. sub-clinical levels of distress. The objective of this study was to examine the psychometric properties of the K10 in Canadian Armed Forces personnel. Data on 6700 Regular Forces personnel were obtained from the 2013 Canadian Forces Mental Health Survey. The internal consistency and factor structure of the K10 (range, 0–40) were examined using confirmatory factor analysis (CFA). Receiver Operating Characteristic (ROC) analysis was used to select optimal cut-offs for the K10, using the presence/absence of any of four past-month disorders as the outcome (posttraumatic stress disorder, major depressive episode, generalized anxiety disorder, and panic disorder). Cronbach’s alpha (0.88) indicated a high level of internal consistency of the K10. Results from CFA indicated that a single-factor 10-item construct had an acceptable overall fit: root mean square error of approximation (RMSEA) = 0.05; 90% confidence interval (CI):0.05–0.06, comparative fit index (CFI) = 0.99, Tucker-Lewis Index (TLI) = 0.99, weighted root mean square residual (WRMR) = 2.06. K10 scores were strongly associated with both the presence and recency of all four measured disorders. The area under the ROC curve was 0.92, demonstrating excellent predictive value for past-30-day disorders. A K10 score of 10 or greater was optimal for screening purposes (sensitivity = 86%; specificity = 83%), while a score of 17 or greater (sensitivity = 53%; specificity = 97%) was optimal for prevalence estimation of clinically significant psychological distress, in that it resulted in equal numbers of false positives and false negatives. Our results suggest that K10 scale has satisfactory psychometric properties for use as a measure of non-specific psychological distress in the military population.

## Introduction

Military personnel are known to have a significant risk of exposure to occupational trauma, which increases their risk of many mental disorders and psychological distress, a phenomenon underlined by the past fifteen years of armed conflict in southwest Asia [1]. Population mental health surveillance data are therefore necessary to guide prevention and control efforts in this at-risk population [2]. One of the key approaches for this crucial task is to administer surveys that include disorder-specific instruments, with a primary focus on conditions of particular concern in the military, notably PTSD, depression, and alcohol use disorders [3]. However, focusing only on the prevalence of specific disorders may underestimate the extent of impaired mental health, given that it is challenging to reliably assess each and every possible disorder in prevalence surveys. There is certainly evidence of a substantial prevalence in military personnel of disorders that have never been systematically assessed in Canadian military research, such as developmental disorders (such as ADHD), intermittent explosive disorder, bipolar disorders, and anxiety disorders such as specific phobias and obsessive-compulsive disorder [4, 5]. Given the practical constraints of assessing many different disorders in health surveys, using brief proxy measures, such as general psychological distress [6], is appealing.

Psychological distress is commonly measured on surveillance surveys using the 10-item Kessler Psychological Distress Scale (K10) measure, which was specifically designed for that purpose [7, 8]. Its developers relied on earlier work suggesting that while measures of psychological distress had items targeting a seemingly diverse set of cognitive, behavioural, emotional, psychophysiological processes, these items tended to load strongly on a single factor conceptualized as “generalized” or “non-specific” psychological distress, which can be contrasted with more specific types of distress related to specific disorders (e.g., distress surrounding obsessions and compulsions in obsessive compulsive disorder) [9, 10]. This important observation opened the door to the creation of a brief, unidimensional measure of generalized psychological distress for use on population health surveys [7]. In their ground-breaking work, Kessler and colleagues developed the K10 through a systematic process in which they first identified 612 candidate items from 18 different instruments, and then gradually reduced these using a variety of techniques (including factor analysis to ensure unidimensionality and item response theory to optimize their measurement of the underlying construct) to come up with the final 10 items in the K10 and its further reduced sibling, the 6-item K6 [7].

The psychometric properties of the K10 (and a further reduced six-item K6) have been extensively examined in civilian populations [11–13]. Exploratory factor analysis has suggested a unidimensional factor structure of the K6 across the general populations of at least 14 nations [14], while similar analysis with the K10 has suggested the possibility of a second factor or even up to four factors [15]. Some investigators have confirmed a single higher order factor using confirmatory factor analysis (CFA), while others found that a model with four factors loading on two first order factors (representing anxiety and depression) fit the data best. There is thus some uncertainty as to the true factor structure of the measures and the extent to which they are invariant across contexts and populations. Nevertheless, treated as a simple scale, the K10 (and K6) consistently show very good reliability as measured by Cronbach’s alpha [12, 16, 17].

The convergent validity evidence of the K10 with other measures of psychological distress (such as the General Health Questionnaire; GHQ) is well-established [8, 17]. Finally, the criterion validity of the K6 and K10 as a tool for predicting serious mental illness is very good to excellent, with the area under the receiver operating characteristic curve (AUC) ranging from 0.76 to 0.85 across 14 nations for the K6 [14] and from 0.87 to 0.88 in two nations for the K10 [7]. Strong predictive value has also generally been noted for past-year mental disorders more broadly (that is, in those with both serious and less serious mental illness) [18], though AUCs as low as 0.71 have been reported [19], and there is some evidence of heterogeneity of AUC values across those of different racial and ethnic backgrounds [19]. The K10 has also been found to correlate with other important mental health-related outcomes [8], such as functional impairment and use of health services.

The K10 is most often treated analytically as a unidimensional scale, which ranges from 0 to 40 or from 10 to 50, depending on the investigator. The remainder of the paper has adjusted results of studies using the 10 to 50 scoring to 0 to 40 scoring to facilitate comparisons. For certain applications (e.g., screening for mental disorders vs. estimation of the prevalence of significant distress) one or more cut-offs have been proposed; these cut-offs vary somewhat depending on the context and the underlying population. The most important consideration in selecting a cutoff is the intended use of the measure. For clinical screening, high sensitivity is essential, and trade-off in terms of lack of specificity can be tolerated provided that subsequent definitive diagnostic steps (e.g., a consultation with a clinician) are not resource-intensive or risky. Screening thresholds will greatly overestimate the prevalence of common mental disorders—a consequence of their low specificity. For example, at a commonly used screening cut-off of 9 or higher, 37.9% of the Canadian general population screened positive, while the prevalence of any mood or anxiety disorder in the population was 15.4% [20]. For epidemiological surveillance of the prevalence of common mental disorders, it may be preferable to have a cut-off that results in roughly an equal number of false positives and false negatives. Such a cut-off results in equal misclassification for those with and without the disease. Consequently, a less sensitive cut-off can be used to more accurately and efficiently determine the prevalence of common mental disorders in the population level or for surveillance purposes. These two types of cut-offs (“screening” vs. “prevalence estimation”) are not interchangeable, justifying the value of determining both within the same population [21]. Others have used multiple cut-offs to split populations into four bands representing low, moderate, high, and very high levels of distress at -points cut of 10, 16, 22, and 30, respectively [22, 23].

Thus, the psychometric properties of the K10 (and K6) have been extensively explored in civilian populations. The measure has also been used for both population health surveillance and/or screening of military personnel in several nations [24–26]. However, documentation of its psychometric properties in military populations is limited. The most extensive validation was undertaken by Blanc and colleagues, who explored its factor structure, internal consistency, concurrent validity, and predictive value for occupational impairments in Canadian military personnel while deployed in Afghanistan [26]. They found that a model with four first-order factors loading on a single higher order factor fit the data much better than a model with just a single higher-order factor (comparative fit indices (CFI) of 0.91 vs. 0.71, respectively) [26]. Internal consistency was very good to excellent (Cronbach’s alpha = 0.89). The K10 showed expected correlations with other commonly used measures of anxiety, depression, and post-traumatic stress. Finally, they noted that K10 score (treated unidimensionally) strongly predicted perceived occupational impairments related to mental health problems (AUC of 0.87), which was similar to the AUC of other measures of anxiety, depression, and post-traumatic stress.

Searle and colleagues explored the internal consistency and predictive value of the K10 for past-30 day and past-year mental disorders assessed using the much more detailed Composite International Diagnostic Interview (CIDI) in the general Australian military population in garrison [21]. They found that the internal consistency was excellent (Cronbach’s alpha = 0.91). Using any of nine past-30 day CIDI affective or anxiety disorders as the outcome, they identified an optimal screening cut-off of 9 or greater (sensitivity = 59%; specificity = 81% and an optimal cut-off for prevalence estimation (which they termed an “epidemiological cut-off”) of 15 or greater (sensitivity = 30%; specificity of 93%).

While these military K10 validation studies have been useful, they provide an incomplete picture of the psychometric properties of the instrument in the general military population. The findings of marginal fit for a unidimensional factor structure is concerning [26], and findings from the general population suggest potential concerns with heterogeneity in terms of its factor structure, optimal cut-offs, and predictive value for mental disorders assessed using more detailed means [27, 28].

Hence, this paper explores the psychometric properties of the K10 in serving Canadian Forces Regular Force personnel using data from the 2013 Canadian Forces Mental Health Survey. Specifically, it 1) assesses its internal structure using Confirmatory Factor Analysis, 2) reports the internal consistency of scores; 3) explores its correlation with outcomes related to mental health (self-rated mental health, disability, mental health services use, and positive mental health) for which a negative correlation is expected for self-rated mental health and positive mental health; 4) presents its association with the presence and recency of common mood, anxiety, and post-traumatic disorders assessed using the CIDI, and 5) using ROC analysis, it explores its overall predictive value and pinpoints optimal cut-offs for screening and for prevalence estimation.

## Methods

### Study population and data source

The Canadian Forces Mental Health Survey was a representative cross-sectional survey of serving Canadian Armed Forces personnel [29]. The target population was all Regular Force personnel as well as Reserve Force personnel who had deployed in support of the mission in Afghanistan who were in uniform in September 2012. The survey employed stratified random sampling framework (with stratification variables being military component (Regular vs. Reserve Force), military rank (in three categories), and Afghanistan mission deployment status (yes or no)) to ensure the representativeness to the Canadian Armed Forces as a whole. The sample size for the survey was determined to achieve a precision of +/-0.7% around a past-year disorder prevalence of 4.0%. Sampled individuals were contacted by Statistics Canada personnel, and data collection occurred from April—August of 2013. The data collection method was via an in-person, computer-assisted interview in the official language of the respondent’s choice (English or French). For most participants, data collection occurred in a private location in the workplace; some participants were interviewed in their homes. Participation in the survey was voluntary, and all participants provided written consent. Ethical review for the data collection occurred within the context of compliance with the relevant Statistics Canada legislation, policies, and directives, including the Statistics Act, as well as its Policy on Privacy and Confidentiality, Directive on Informing Survey Respondents, Directive on Record Linkage, and the Directive on Security of Sensitive Statistical Information. After compliance was demonstrated, the survey was approved by Canada`s Chief Statistician. The particular analysis in the present paper was approved through Statistics Canada`s Federal Research Data Centre Program (https://www.statcan.gc.ca/eng/rdc/frdc_application_process_guidelines), which is responsible for ensuring the scientific merit and compliance with ethical and other standards. This approval process involves review by the Senior Statistical Focal Point for the Department of National Defence, as well as Statistics Canada`s Departmental Audit Committee, and the Director of Subject Matter for the particular theme of the proposed analysis. Data were obtained from Statistics Canada, and the authors had no access to any potentially identifying participant information. More detailed information about the methodology of the 2013 Canadian Forces Mental Health Survey is available elsewhere [1]. The analyses of the present study are restricted to the Regular Force sample (N = 6,700; with a response rate of 80%), representative of approximately 68,000 Regular Force personnel.

### Measures

#### Sociodemographic and military characteristics of respondents

Sample characteristics were described using administrative data from the CAF (age, sex, element [Army, Navy, or Air Force], and rank category or via survey items developed by Statistics Canada for its household surveys (language of interview, marital status, educational attainment, and household income).

#### Psychological distress

Psychological distress was measured using the 10-item K10 scale, which is described in detail in the Introduction. Responses were scored on a five-point ordinal scale reflecting how much of over the past month time respondents had experienced 10 symptoms, such as “feeling tired out for no good reason” and “sad or depressed”. The measure has five response categories ranging from 0 (none of the time) to 4 (all of the time). The items were summed to generate a total score ranging from 0 to 40, with higher scores indicating higher levels of psychological distress.

#### Self-rated mental health

Self-rated mental health was assessed using a single item: “In general, would you say your mental health is…”, with response options of poor, fair, good, very good, and excellent. The item was treated as a scale variable with poor = 1 and excellent = 5, so a higher score indicated better self-rated mental health.

#### Disability

Disability across six life domains was assessed using the World Health Organization Disability Assessment Schedule, Version 2 (WHODAS-2), using the “complex” scoring algorithm. WHODAS-2 scores range from 0–100, with higher scores reflecting *greater* disability. The WHODAS-2 has undergone validation in general population and in military samples [30].

#### Past-year professional mental health services use

Past-year professional mental health services use was assessed using the “Services” module of the Composite International Diagnostic Interview (CIDI). Respondents were asked: “During the past 12 months, have you seen, or talked on the telephone to, any of the following people about problems with your emotions, mental health or use of alcohol or drugs?”, with response options of psychiatrist, psychologist, general practitioner or family doctor, nurse (including a case manager), or social worker, counselor, or psychotherapist. Those responding affirmatively to any of these were categorized as having any past-year professional mental health services use.

#### Positive mental health

Positive mental health was assessed using the Mental Health Continuum, Short-form (MHC-SF), a 14-item scale assessing three dimensions of positive mental health (positive emotional, psychological, and social well-being [31]. The items assess how often, over the past month, the respondent had felt… (happy, that people are basically good, that [they] had warm and trusting relationships with others, etc.), with response categories of never, once or twice, about once a week, about two or three times a week, almost every day, and every day. The items were scored from 0 (never) to 5 (every day) and then summed (range, 0–70), with higher scores representing greater positive mental health. The MHC-SF has been validated in general populations but not, to our knowledge, in military populations. It demonstrated a Cronbach’s alpha of 0.90 and an ordinal alpha of 0.99 on the current data, indicating a high level of internal consistency.

#### Mental disorders

Mental disorders were assessed with the World Health Organization World Mental Health Composite International Diagnostic Interview (WMH-CIDI), which is a comprehensive, structured lay-administered psychiatric interview for the assessment of mental disorders [32]. WMH-CIDI has shown good consistency with clinical diagnostic instruments [33, 34]. The mental disorders measured include: posttraumatic stress disorder (PTSD), major depressive episode (MDE), generalized anxiety disorder (GAD), and panic disorder (PD)”; The DSM-IV criteria were used to create an algorithm to classify people as having the mental health conditions. WMH-CIDI identified individuals who had ever met criteria for mental disorders in their lifetime (*lifetime disorders*) or during the past 12 months (*past-year disorders*). We have also identified those with a WMH-CIDI diagnosed lifetime disorder but no past-year disorder (*remote disorders*), those with past-year disorder but no past-month mood and anxiety disorder (*recent disorders*), and those with past-month mood and anxiety disorder (*current disorders*). For those with a past-year disorder, recency was assessed using a single item in each of the disorder-specific modules that asked how recently an episode of the key features of the disorder (e.g., a period of low mood or anhedonia in those with depression) had occurred, with options including the past month, two to six months previously, and more than six months previously. One aggregate variable was constructed indicating those with any mood or anxiety disorder (PTSD, MDE, GAD, or PD). For this indicator, “*never*” stands for never having had any of the four lifetime disorders; “*remote*” for those who had at least one lifetime disorder but no past-year disorders; “*recent*” for those who had at least one past-year disorder but no 30-day disorder; and “*current*” for individuals who had at least one 30-day disorder.

### Statistical analysis

**Fig 1:**
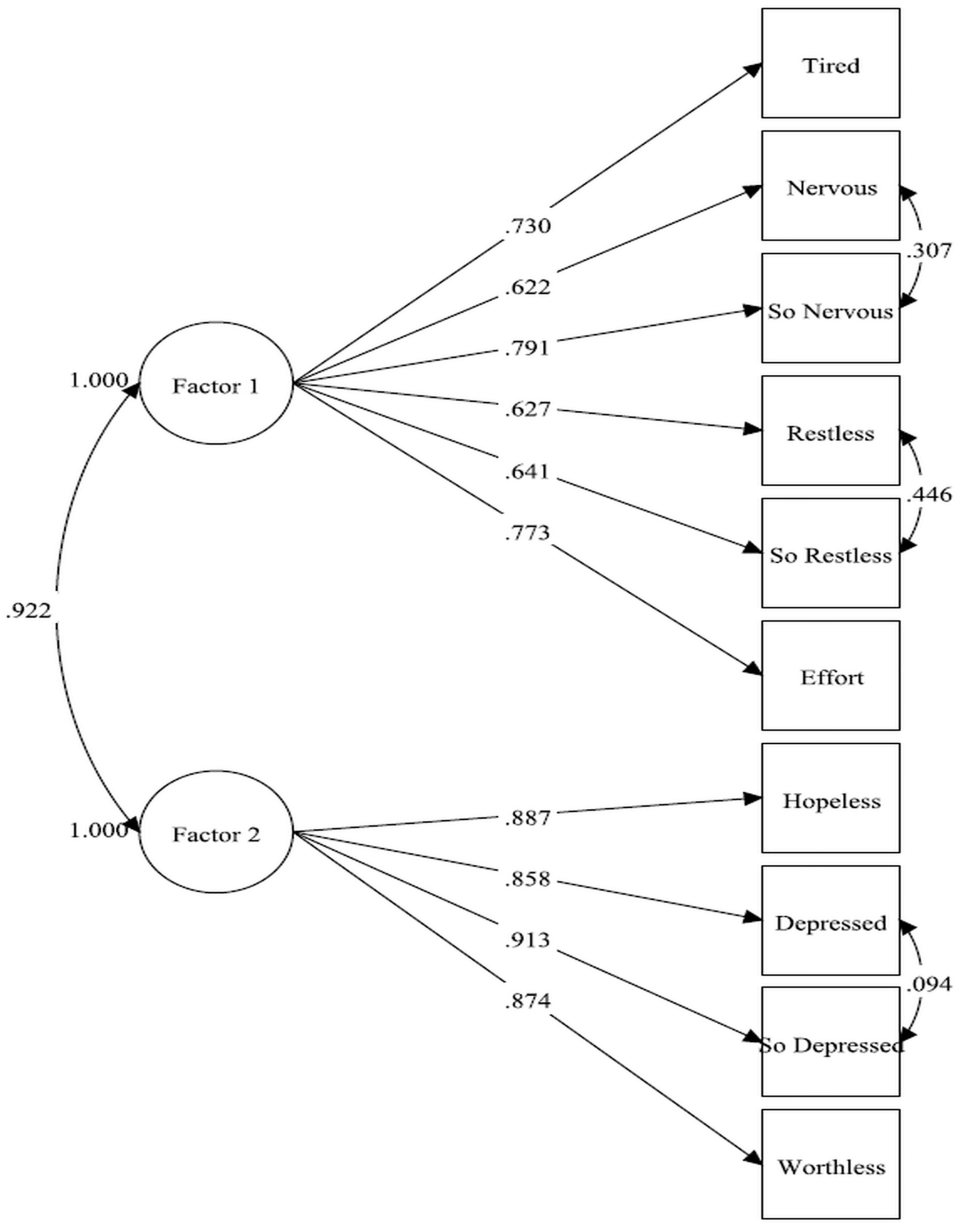
Model 2 for confirmatory factor analysis of K10 items. Note: Factor 1 = Anxiety; Factor 2 = Depression.

The factor structure of the K10 was examined via confirmatory factor analysis (CFA) using structural equation modeling on the K10 items (treated as ordinal variables and hence using the polychoric correlation matrix and MPlus’ WLSMV estimator). We explored two competing models based on Blanc and colleague’s CFA work on another Canadian military sample [26]. Specifically, Model 1 had all 10 items loading on a single higher-order factor representing general distress. Model 2 had the items loading on two correlated first-order factors representing depression and anxiety (Fig 1).

The version of the K10 used on the survey included three items with skip patterns. For example, one item asked how often the respondent had been “nervous”, and the next item then asked how often the respondent had been “so nervous that nothing could calm you down.” Those answering “never” to the first item would skip the second item, with a response of “never” (score = 0) being reasonably imputed for it. To address these interdependencies, we specified correlated error terms for the three pairs of items with such skip patterns in both of our models [12]. Goodness of fit was determined using absolute (i.e., chi-square and root-mean-square error of approximation (RMSEA)) and comparative (i.e. comparative fit index (CFI) and Tucker-Lewis Index (TLI)) fit indices. Model fit was considered to be acceptable if RMSEA is equal or lower than 0.08, CFI and TLI are equal or greater than 0.95, and if WRMR is equal or greater than 0.90 [35]. However, we did not rely on the chi-square test to examine model fit because current analyses used a large sample size, and chi-square is sensitive to sample size [36]. Internal consistency of the K10 items was tested using Cronbach’s alpha (treating the items as scale variables) and confirmed using an ordinal alpha that addressed potential violations of assumptions, including normality and lack of correlated errors. Given the construct-confirming nature of the CFA, these analyses were unweighted [37].

The remaining analyses were completed with SPSS version 24.0 (IBM Corp., Armonk, NY), and all were weighted to make the results representative of the underlying population. The association of the K10 score with mental health outcomes were explored using Pearson correlation coefficients (r) for disability, positive mental health, and self-rated mental health and point-biserial correlations (r_pb_) for past-year mental health services use. The association of the K10 score with the presence and recency of the four mood, anxiety, and post-traumatic disorders assessed by the survey was examined using the mean value of the K10 in those with current, recent, and remote disorders (as defined above). In keeping with Statistics Canada’s confidentiality restrictions for this survey, unweighted cell counts are not reported, values pertaining to a cell with an unweighted count of less than 5 are suppressed, and all proportions were calculated using weighted cell counts rounded to the nearest 20.

Receiver Operating Characteristic (ROC) analysis was used to select optimal cut-offs for the K10 [38, 39], using the presence/absence of past-month mood and anxiety disorders as the outcome. Prevalence-dependent and prevalence-independent ROC indices were calculated manually from weighted contingency table data, using proportions calculated from rounded, weighted cell counts. AUC was estimated non-parametrically using SPSS’s *ROC Curve* analysis command; this required rescaling of the fractional sampling weights to convert them to integers to accommodate the non-parametric approach. Confidence intervals (CI) for weighted analyses were estimated using a manual bootstrap approach with 500 bootstrap replicate weights provided by Statistics Canada. Non-overlapping 95% CIs indicate statistically significant differences [40]. In accordance with Statistics Canada’s data quality standards, estimates with high coefficient of variation (16.5–33.3%) are flagged, and those with a coefficient of variation of more than 33.3% are suppressed.

Following the approach of a previous study that examined the validity of K10 in Australian military personnel [21], we examined both a “screening” and “prevalence estimation” cut-off. The screening cut-off was explored using the Youden index [41]. This index is equivalent to maximization of the sum of sensitivity and specificity [42, 43]. We also explored the extent of misclassification at a given cut-off using contingency tables, with the optimal prevalence estimation cut-off being one in which the extent of misclassification was the same in both directions (that is, that the number of false positives and false negatives are closest together) [21]. Cases representing less than 0.1% were excluded due to missing data on K10 score.

Missing data for other variables were handled through complete case analysis.

## Results

### Participant characteristics

Table 1 presents demographic and military characteristics of the sample. Respondents were predominantly young and middle-aged, male, and married or in common law relationships. More than half had a post-secondary degree and a household income of $80,000 or more. Slightly more than half were in the Army and were junior non-commissioned members (NCMs).

**Table 1:** Sociodemographic and military characteristics.

	Weighted N	Weighted %	95% CI
**Age**			
24 years or less	8560	13.3	12.4–14.2
25–34 years	24220	37.6	36.4–38.8
35–44 years	17860	27.7	26.6–28.9
45 years or more	13760	21.4	20.4–22.3
**Sex**			
Male	55480	86.1	85.4–86.9
Female	8920	13.9	13.1–14.6
**Marital status**			
Married or common law	42200	65.6	64.4–66.7
Separated, divorced, or widowed	4840	7.5	6.9–8.1
Never married	17300	26.9	25.7–28.1
**Education**			
Secondary graduation or less	19160	29.8	24.6–26.9
Some post—secondary	5700	8.9	8.1–9.7
Post—secondary graduation	39400	61.3	60.1–62.5
**Household income**			
None or less than $40,000	2300	3.6	2.1–2.9
$40,000-$59,000	6060	9.4	8.6–10.2
$60,000-$79,000	14600	22.7	21.5–23.8
$80,000 or more	41440	64.3	63.2–65.5
**Language of interview**			
English	50600	78.6	77.6–79.6
French	13800	21.4	21.4–21.4
**Element**			
Army	34220	53.1	52–54.3
Navy	11080	17.2	16.2–18.2
Air	19100	29.7	28.5–30.8
**Rank**			
Junior NCM	35440	55.0	54.8–55.2
Senior NCM	15500	24.1	23.8–24.3
Officer	13460	20.9	19.9–21.9

CI: confidence interval; NCM: noncommissioned member.

Senior NCM includes the rank of sergeant (and equivalents in other environments) through chief warrant officer.

### Confirmatory factor analysis (CFA) and internal consistency

Results from the CFAs for two competing models are presented in Table 2. Model 1 had all 10 items loading on a single higher-order generalized distress factor, whereas Model 2 had the 10 items loading on 2 highly-correlated first-order factors representing anxiety and depression, respectively (Fig 1). In absolute terms, both models had adequate fit across all fit indices, as defined in the Methods section. However, the more parsimonious single factor model (Model 1) fit almost as well (RMSEA = 0.05 and 90% CI: 0.05–0.06, CFI = 0.99, TLI = 0.99, WRMR = 2.06) and thus retained for subsequent analyses. The K10 items demonstrated a Cronbach’s alpha of 0.88 and an ordinal alpha of 0.92, indicating a high level of internal consistency.

**Table 2:** Fit indices for confirmatory factor analyses.

Fit Index	Criterion of Fit	Index Value		Fit/No Fit	
		Model 1	Model 2	Model 1	Model 2
Χ2 (df)	p ≥ 0.05	685.37 (32) (p<0.001)	393.44 (31) (p<0.001)	^a^	^a^
RMSEA (90% CI)	≤ 0.08	0.05 (0.05–0.06)	0.04 (0.04–0.05)	Fit	Fit
CFI	≥ 0.95	0.99	0.99	Fit	Fit
TLI	≥ 0.95	0.99	0.99	Fit	Fit
WRMR	≥0.90	2.06	1.50	Fit	Fit

Model 1 = All 10 items loading on a single higher-order factor.

Model 2 = 10 items loading on two correlated first-order factors (Fig 1)

DF: degree of freedom; RMSEA: root mean square error of approximation; CI: confidence interval; CFI: comparative fit index; TLI: Tucker-Lewis Index; WRMR: weighted root mean square residual.

^a^The large sample sizes in this study precludes the use of the chi-square fit measure, because chi-square is sensitive to sample size.

### K10 and outcomes known to be related to distress and mental disorders

K10 had the expected positive correlation with outcomes known to be related to distress and mental disorders, including disability (r = 0.63; 95% CI: 0.62–0.65) and past-year mental health services use (r = 0.57; 95% CI: 0.55–0.60). In contrast, the K10 score had the expected negative correlation with self-rated mental health (r = -0.43; 95% CI: -0.40 –-0.45) and positive mental health (r_pb_ = -0.60; 95% CI: -0.62 –-0.58).

### K10 and the presence and recency of mental disorders

K10 distress was consistently related to the presence and recency of MDE, PTSD, GAD, PD, and any of the foregoing disorders (Table 3). Individuals with current (past-month) disorders had the highest levels of distress, followed by those with recent disorders. These were all followed much more distantly by those with remote disorders, though distress levels in those with remote disorders were significantly higher than those without any past disorders.

**Table 3:** K10 scores as a function of mental disorder recency (overall N = 64340).

Disorder	Recency	N, % (95% CI)	Mean K10 score (95% CI)
MDE	Never	54120, 84.4% (83.5–85.3)	5.49 (5.37–5.62)
	Remote	4960, 7.7% (7.1–8.4)	7.37 (6.94–7.81)
	Recent	3100, 4.8% (4.3–5.4)	13.46 (12.67–14.25)
	Current	1960, 3.1% (2.6–3.5)	21.01 (20.03–21.99)
	Total	64140	6.5 (6.35–6.64)
PTSD	Never	56420, 88.9% (88.1–89.7)	5.74 (5.6–5.87)
	Remote	3700, 5.8% (5.3–6.4)	8.91 (8.31–9.5)
	Recent	1300, 2% (1.7–2.4)	14.01 (12.91–15.11)
	Current	2040, 3.2% (2.8–3.7)	17.47 (16.41–18.52)
	Total	63460	6.47 (6.33–6.61)
GAD	Never	56220, 88% (87.2–88.8)	5.59 (5.46–5.72)
	Remote	4720, 7.4% (6.7–8)	10.3 (9.76–10.85)
	Recent	1460, 2.3% (1.9–2.7)	15.95 (14.85–17.06)
	Current	1500, 2.3% (2–2.7)	18.71 (17.55–19.88)
	Total	63900	6.48 (6.34–6.63)
PD	Never	59600, 94.2% (93.6–94.8)	6.03 (5.9–6.17)
	Remote	1540, 2.4% (2.1–2.8)	10.61 (9.61–11.62)
	Recent	1180, 1.9% (1.5–2.2)	15.32 (14.04–16.59)
	Current	940, 1.5% (1.2–1.8)	19.52 (17.65–21.39)
	Total	63260	6.52 (6.37–6.66)
Any, mood, anxiety, or posttraumatic disorder	Never	45320, 72.2% (71.1–73.3)	4.71 (4.6–4.83)
	Remote	8940, 14.2% (13.4–15.1)	7.73 (7.39–8.07)
	Recent	4060, 6.5% (5.9–7.1)	12 (11.4–12.6)
	Current	4420, 7% (6.4–7.7)	17.61 (16.91–18.31)
	Total	62760	6.52 (6.38–6.67)

Note: K10 = Kessler 10; CI = confidence interval; Remote = lifetime but not in past year; Recent = past-year but not in past month; Current = in past month; MDE = major depressive episode; PTSD = posttraumatic stress disorder; GAD = generalized anxiety disorder; PD = panic disorder. See methods for coding of any disorder variable. Totals differ due to differing amounts of missing data by disorder. Cases representing less than 0.1% were excluded due to missing data on K10 score.

### ROC analysis

**Fig 2:**
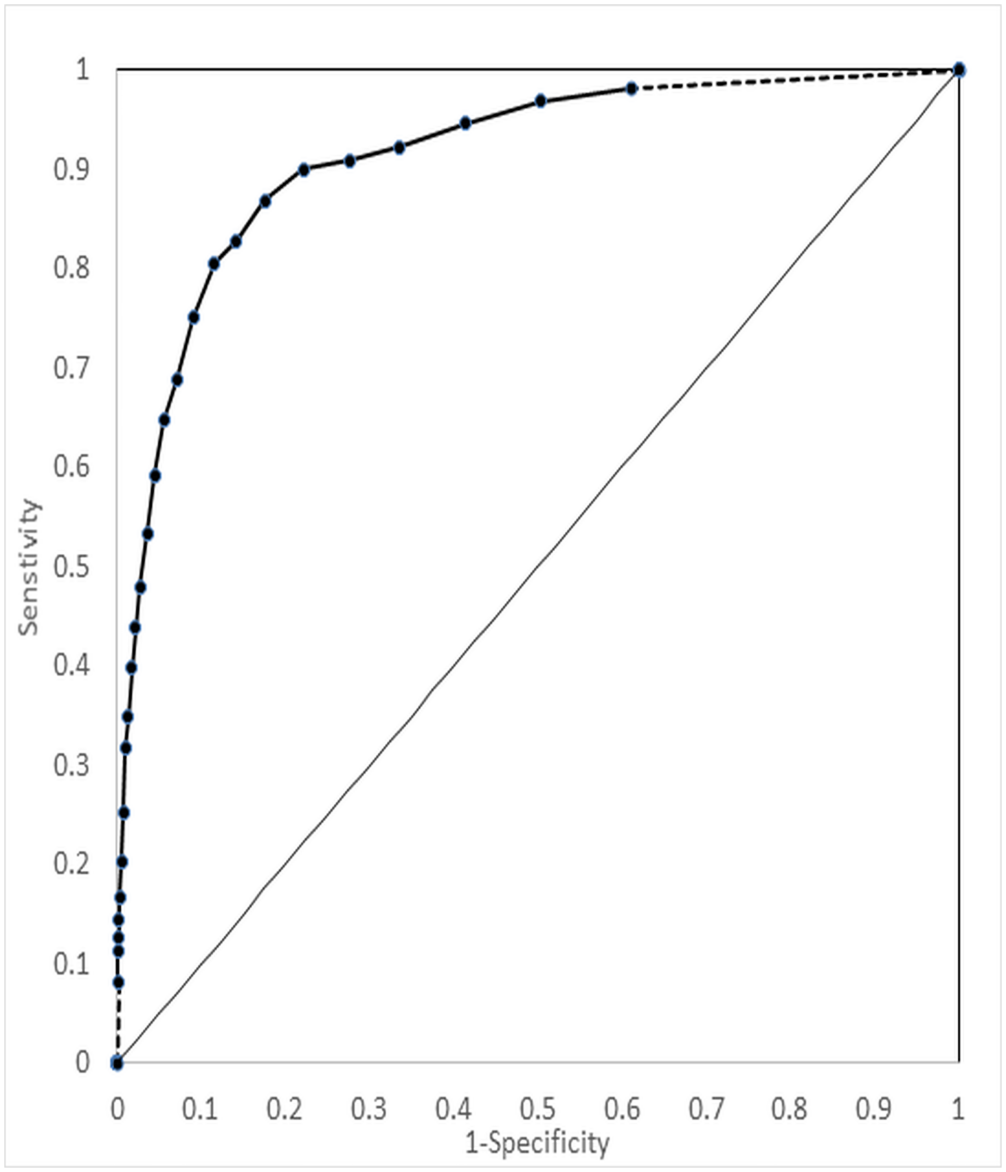
ROC curve for past-month mood/anxiety/posttraumatic disorder as a function of K10 score. Note: Coordinates suppressed for cut-offs <4 or > 29 to comply with Statistics Canada’s release guidelines related to small cell sizes. Area under the ROC curve (AUC) = 0.915; 95% CI: 0.900–0.929.

Fig 2 illustrates the ROC curve using any past-month disorder as the reference. The K10 had an AUC of 0.915 (95% CI, 0.900–0.929), showing that K10 score was a strong predictor of the outcome of any past-month disorder. The ROC analysis results for prevalence-dependent and prevalence-independent indices are shown in Tables 4 and 5, respectively. Scanning the indices across the potential cut-offs, no clear K10 score thresholds are apparent with respect to most of the indices. For % correctly classified and specificity, however, the values plateau around a cut-off of 18 or so, beyond which little improvement is seen.

**Table 4:** ROC analysis of K10 score as a predictor of any past-month disorder: Prevalence-dependent indices (overall N = 62280).

K10 Cut-off	Proportion at or above cut-off (95% CI)	False negative proportion (95% CI)	False positive proportion (95% CI)	Correctly classified proportion (95% CI)	Positive predictive value (95% CI)	Negative predictive value (95% CI)
4	0.636 (0.623–0.649)	0.001 (0–0.002)	0.567 (0.554–0.58)	0.432 (0.419–0.445)	0.109 (0.099–0.119)	0.996 (0.994–0.998)
5	0.535 (0.521–0.549)	0.002 (0.001–0.003)	0.467 (0.453–0.481)	0.531 (0.517–0.545)	0.128 (0.116–0.14)	0.995 (0.992–0.998)
6	0.451 (0.438–0.464)	0.004 (0.002–0.006)	0.384 (0.371–0.397)	0.612 (0.599–0.625)	0.149 (0.135–0.163)	0.993 (0.99–0.996)
7	0.378 (0.365–0.391)	0.005 (0.003–0.007)	0.312 (0.299–0.325)	0.683 (0.67–0.696)	0.173 (0.157–0.189)	0.991 (0.988–0.994)
8	0.322 (0.309–0.335)	0.006 (0.004–0.008)	0.257 (0.245–0.269)	0.737 (0.725–0.749)	0.2 (0.182–0.218)	0.991 (0.988–0.994)
9	0.27 (0.258–0.282)	0.007 (0.005–0.009)	0.206 (0.195–0.217)	0.787 (0.776–0.798)	0.237 (0.216–0.258)	0.99 (0.987–0.993)
10	0.225 (0.214–0.236)	0.009 (0.006–0.012)	0.163 (0.154–0.172)	0.827 (0.817–0.837)	0.274 (0.25–0.298)	0.988 (0.985–0.991)
11	0.191 (0.181–0.201)	0.012 (0.009–0.015)	0.132 (0.123–0.141)	0.856 (0.847–0.865)	0.307 (0.28–0.334)	0.985 (0.981–0.989)
12	0.165 (0.156–0.174)	0.014 (0.011–0.017)	0.107 (0.099–0.115)	0.879 (0.871–0.887)	0.347 (0.317–0.377)	0.983 (0.979–0.987)
13	0.137 (0.128–0.146)	0.018 (0.015–0.021)	0.084 (0.077–0.091)	0.898 (0.89–0.906)	0.388 (0.355–0.421)	0.98 (0.976–0.984)
14	0.115 (0.107–0.123)	0.022 (0.018–0.026)	0.066 (0.06–0.072)	0.912 (0.905–0.919)	0.423 (0.386–0.46)	0.975 (0.971–0.979)
15	0.098 (0.09–0.106)	0.025 (0.021–0.029)	0.052 (0.046–0.058)	0.923 (0.916–0.93)	0.469 (0.429–0.509)	0.972 (0.967–0.977)
16	0.083 (0.076–0.09)	0.029 (0.024–0.034)	0.041 (0.036–0.046)	0.93 (0.923–0.937)	0.506 (0.463–0.549)	0.969 (0.964–0.974)
**17**	**0.071** **(0.065–0.077)**	**0.033** **(0.028–0.038)**	**0.033** **(0.028–0.038)**	**0.934** **(0.927–0.941)**	**0.532** **(0.485–0.579)**	**0.964** **(0.959–0.969)**
18	0.059 (0.053–0.065)	0.037 (0.032–0.042)	0.025 (0.021–0.029)	0.938 (0.932–0.944)	0.573 (0.521–0.625)	0.961 (0.956–0.966)
19	0.052 (0.046–0.058)	0.04 (0.035–0.045)	0.02 (0.016–0.024)	0.94 (0.934–0.946)	0.602 (0.547–0.657)	0.958 (0.953–0.963)
20	0.045 (0.04–0.05)	0.043 (0.038–0.048)	0.016 (0.013–0.019)	0.941 (0.935–0.947)	0.633 (0.576–0.69)	0.955 (0.95–0.96)
21	0.037 (0.032–0.042)	0.046 (0.041–0.051)	0.012 (0.009–0.015)	0.942 (0.936–0.948)	0.675 (0.616–0.734)	0.952 (0.946–0.958)
22	0.032 (0.027–0.037)	0.048 (0.042–0.054)	0.009 (0.007–0.011)	0.942 (0.936–0.948)	0.707 (0.644–0.77)	0.95 (0.944–0.956)
23	0.025 (0.021–0.029)	0.053 (0.047–0.059)	0.007 (0.005–0.009)	0.94 (0.934–0.946)	0.718 (0.646–0.79)	0.946 (0.94–0.952)
24	0.019 (0.015–0.023)	0.056 (0.05–0.062)	0.005 (0.003–0.007)^E^	0.939 (0.933–0.945)	0.75 (0.671–0.829)	0.942 (0.936–0.948)
25	0.014 (0.011–0.017)	0.059 (0.053–0.065)	0.003 (0.002–0.004)^E^	0.938 (0.932–0.944)	0.822 (0.738–0.906)	0.94 (0.934–0.946)
26	0.013 (0.01–0.016)	0.06 (0.054–0.066)	0.002 (0.001–0.003)^E^	0.937 (0.931–0.943)	0.821 (0.735–0.907)	0.939 (0.933–0.945)
27	0.011 (0.008–0.014)	0.062 (0.056–0.068)	0.002 (0.001–0.003)^E^	0.936 (0.93–0.942)	0.848 (0.752–0.944)	0.937 (0.931–0.943)
28	0.009 (0.006–0.012)	0.063 (0.057–0.069)	^F^	0.936 (0.93–0.942)	0.862 (0.762–0.962)	0.937 (0.931–0.943)
29	0.006 (0.004–0.008)^E^	0.065 (0.059–0.071)	0.001 (0.001–0.001)^E^	0.934 (0.928–0.94)	0.947 (0.868–1.026)	0.934 (0.928–0.94)

Note: CI = confidence interval; optimal cut-off for prevalence estimation (where false positive proportion = false negative proportion) is show in **bold**. Area under the ROC curve (AUC) = 0.915; 95% CI, 0.900–0.929. Indices for cut-offs < 4 or >29 are suppressed due to Statistics Canada’s vetting requirements related to small cell sizes. Cases representing 3.3% of the population are missing due to missing K10 or any past-month disorder variables.

^E^ Coefficient of variation > 16.5% and < = 33.3%; high level of sampling error.

^F^ Values suppressed due to extreme level of sampling error (coefficient of variation > 33.3%).

**Table 5:** ROC analysis of K10 score as a predictor of any past-month disorder: Prevalence-independent indices (overall N = 62280).

Cut-off	Proportion at or above cut-off [95% CI]	Sensitivity [95% CI]	Specificity [95% CI]	Youden Index [95% CI]	Positive Likelihood Ratio [95% CI]	Negative Likelihood Ratio [95% CI]
4	0.636 (0.623–0.649)	0.982 (0.97–0.994)	0.39 (0.377–0.404)	0.372 (0.354–0.391)	1.6 (1.6–1.7)	^F^
5	0.535 (0.521–0.549)	0.968 (0.951–0.985)	0.498 (0.483–0.512)	0.466 (0.444–0.488)	1.9 (1.9–2)	0.06 (0.03–0.1)^E^
6	0.451 (0.438–0.464)	0.946 (0.921–0.97)	0.587 (0.573–0.601)	0.533 (0.504–0.561)	2.3 (2.2–2.4)	0.09 (0.05–0.13)^E^
7	0.378 (0.365–0.391)	0.923 (0.896–0.95)	0.664 (0.65–0.678)	0.587 (0.557–0.618)	2.7 (2.6–2.9)	0.12 (0.08–0.16)^E^
8	0.322 (0.309–0.335)	0.91 (0.881–0.938)	0.723 (0.711–0.735)	0.633 (0.601–0.664)	3.3 (3.1–3.5)	0.13 (0.09–0.16)
9	0.27 (0.258–0.282)	0.9 (0.871–0.929)	0.778 (0.767–0.79)	0.679 (0.647–0.71)	4.1 (3.8–4.3)	0.13 (0.09–0.17)
**10**	**0.225** **(0.214–0.236)**	**0.869** **(0.836–0.902)**	**0.824** **(0.814–0.834)**	**0.693** **(0.659–0.728)**	**4.9** **(4.6–5.3)**	**0.16** **(0.12–0.2)**
11	0.191 (0.181–0.201)	0.828 (0.792–0.864)	0.858 (0.848–0.867)	0.686 (0.648–0.724)	5.8 (5.4–6.3)	0.2 (0.16–0.24)
12	0.165 (0.156–0.174)	0.805 (0.768–0.843)	0.884 (0.876–0.893)	0.69 (0.651–0.729)	7 (6.3–7.6)	0.22 (0.18–0.26)
13	0.137 (0.128–0.146)	0.751 (0.709–0.794)	0.91 (0.902–0.917)	0.661 (0.617–0.704)	8.3 (7.5–9.2)	0.27 (0.23–0.32)
14	0.115 (0.107–0.123)	0.688 (0.642–0.734)	0.929 (0.922–0.936)	0.617 (0.57–0.663)	9.7 (8.5–10.8)	0.34 (0.29–0.39)
15	0.098 (0.09–0.106)	0.647 (0.6–0.694)	0.944 (0.938–0.95)	0.591 (0.544–0.638)	11.6 (10–13.1)	0.37 (0.32–0.42)
16	0.083 (0.076–0.09)	0.593 (0.545–0.64)	0.956 (0.95–0.961)	0.549 (0.501–0.596)	13.4 (11.4–15.4)	0.43 (0.38–0.48)
17	0.071 (0.065–0.077)	0.534 (0.485–0.583)	0.964 (0.959–0.969)	0.498 (0.448–0.548)	14.9 (12.4–17.4)	0.48 (0.43–0.53)
18	0.059 (0.053–0.065)	0.48 (0.43–0.529)	0.973 (0.969–0.977)	0.453 (0.403–0.502)	17.8 (14.3–21.3)	0.53 (0.48–0.59)
19	0.052 (0.046–0.058)	0.439 (0.39–0.488)	0.978 (0.974–0.982)	0.417 (0.368–0.466)	20.2 (15.9–24.5)	0.57 (0.52–0.62)
20	0.045 (0.04–0.05)	0.398 (0.351–0.445)	0.982 (0.979–0.986)	0.381 (0.334–0.427)	22.6 (17.3–27.9)	0.61 (0.56–0.66)
21	0.037 (0.032–0.042)	0.348 (0.302–0.395)	0.987 (0.984–0.99)	0.336 (0.289–0.382)	27.3 (19.8–34.7)	0.66 (0.61–0.71)
22	0.032 (0.027–0.037)	0.317 (0.27–0.363)	0.99 (0.987–0.993)	0.307 (0.261–0.353)	31.6 (21.4–41.9)	0.69 (0.64–0.74)
23	0.025 (0.021–0.029)	0.253 (0.209–0.298)	0.992 (0.99–0.995)	0.246 (0.201–0.291)	33.4 (20.9–45.8)^E^	0.75 (0.71–0.8)
24	0.019 (0.015–0.023)	0.204 (0.163–0.244)	0.995 (0.993–0.997)	0.198 (0.158–0.239)	39.3 (20–58.6)^E^	0.8 (0.76–0.84)
25	0.014 (0.011–0.017)	0.167 (0.13–0.205)	0.997 (0.996–0.999)	0.165 (0.128–0.202)	^F^	0.83 (0.8–0.87)
26	0.013 (0.01–0.016)	0.145 (0.11–0.179)	0.998 (0.997–0.999)	0.143 (0.108–0.177)	^F^	0.86 (0.82–0.89)
27	0.011 (0.008–0.014)	0.127 (0.092–0.161)	0.998 (0.997–0.999)	0.125 (0.091–0.159)	^F^	0.87 (0.84–0.91)
28	0.009 (0.006–0.012)	0.113 (0.08–0.146)	0.999 (0.997–1)	0.112 (0.079–0.145)	^F^	0.89 (0.86–0.92)
29	0.006 (0.004–0.008)^E^	0.081 (0.053–0.109)^E^	0.999 (0.999–1)	0.081 (0.053–0.109)^E^	^F^	0.92 (0.89–0.95)

Note: CI = confidence interval; optimal cut-off for screening (where the Youden index is maximized) is show in **bold**. Area under the ROC curve (AUC) = 0.915; 95% CI, 0.900–0.929. Indices for cut-offs <4 or >29 are suppressed due to Statistics Canada’s vetting requirements related to small cell sizes. Cases representing 3.3% of the population are missing due to missing K10 score or any past-month disorder variables.

^E^ Coefficient of variation > 16.5% and < = 33.3%; high level of sampling error.

^F^ Values suppressed due to extreme level of sampling error (coefficient of variation > 33.3%).

For the purpose of estimation of the prevalence of any of the four past-month disorders assessed, an optimal cut off of 17 or greater (seen in 7.1% of the population) is identified in that the false positive and false negative proportions are closest to equal (3.3% each; Table 5). The Youden Index is maximized at a cut-off of 10 or greater (Table 5), which thus corresponds to an optimal screening cut-off. At that cut-off, the sensitivity and specificity are 0.869 and 0.824, respectively. At the outcome prevalence seen in our population (7.0%), the PPV at the screening cut-off is modest (0.274) but the NPV is excellent (0.988). At the screening cut-off, the positive likelihood ratio was 4.9 and the negative likelihood ratio was 0.16, reflecting very good discriminatory power at that cut-off.

## Discussion

### Summary of key findings

In this paper, we sought to explore the psychometric properties of a widely-used general psychological distress scale, the K10. Using CFA, we confirmed that a model with all ten items loading on a single higher-order fit the data adequately across all fit indices, using *a priori* values for adequacy of fit. However, a model in which the ten items loaded on two first-order factors (representing anxiety and depression) fit the data slightly better. The K10 scale had excellent internal consistency (Cronbach’s alpha = 0.88). K10 scores were positively correlated with other outcomes related to distress and mental disorders, including self-rated mental health, disability, and past-year mental health services use; it was negatively correlated with positive mental health. K10 scores were significantly higher in those with MDE, PTSD, GAD, and PD (and an aggregate outcome of any of the foregoing). K10 scores were also much higher in those with more recent disorders. In ROC analysis, the K10 was a strong predictor of the outcome of any past-month disorder (AUC = 0.914). A cut-off of 17 or greater proved optimal for the purposes of estimation of the prevalence of past-month mood, anxiety, and post-traumatic disorders. In contrast, a cut-off of 10 or higher was identified as optimal for screening purposes.

### Comparison with other relevant literature

The K10 was explicitly designed to be a unidimensional measure of generalized psychological distress, and most analyses have indeed found (as we did) a single-factor structure using factor analytic techniques and favourable internal consistency [27, 28]. Our findings cohere with those of Bougie et al. (2016), in that we found that a more complex model with two first-order factors loading fit the data slightly better—though the strong correlation between the two factors (0.92) suggested that it offered little conceptual advantage over a more parsimonious single-factor model. Others have also documented the expected association of K10 scores with self-rated mental health, disability, past-year mental health services use, and positive mental health [28, 44, 45].

A number of different cut-offs have been proposed for the K10 [17, 21, 26, 28], depending on the population studied, the outcome against which it was validated (and how it was measured), and the goal of the cut-off (e.g., for clinical screening vs. estimation of the prevalence of distress in population health surveys) [21]. The most comparable work in a military population comes from the Australian Defence Force, in which a slightly lower cut-off of 15 (converted to the 0–40 scaling we used) was deemed optimal for prevalence estimation [21]. However, their cut-off showed a sensitivity (0.30) that was lower than ours (0.53). Our optimal screening cut-off of 10 is also similar to their screening cut-off of 9 (converted to the 0–40 scaling we used). Their screening cut-off again showed a lower sensitivity 0.59 than ours (0.87). It is possible that the poor final response rate for the Australian survey (24%) and the use of different outcome measures (a broader range of mood and anxiety disorders assessed using ICD-10 criteria) explain the observed discrepancies. Blanc et al. (2014) instead proposed a screening cut-off of 6 (converted to the 0–40 scaling we used, based on the maximum of sum of sensitivity and specificity) in a convenience sample of CAF personnel serving in Afghanistan [26]. Differences in the study population, the context (a combat deployment), and the outcome (the presence of occupational impairment as opposed to past-month mood and anxiety disorders) likely account for the differences in that finding from our own. However, Cornelius et al. (2013) proposed an optimal screening cut-off of 14 for K10 (converted to the 0–40 scaling), using past-month DSM-IV mental disorders including any mood, anxiety, or substance use disorders (assessed by CIDI) in a general population sample [17]. Fassaert et al. (2009) documented cut-off scores of 12.5 for Moroccan and Turkish participants and 6.5 for ethnic Dutch participants for K10 (converted to the 0–40 scaling), using one-month DSM-IV diagnosis for depressive and/or anxiety disorder [28]. These findings on differing cut-offs demonstrate the importance of exploring the psychometric properties of the K10 in a given target population.

### Limitations and strengths

This study has several limitations. First, we were limited to exploring the predictive value of the K10 for the four mood, anxiety, and post-traumatic disorders assessed with the disorder-specific modules of the CIDI. A number of other mental disorders have been shown to be prevalent in military personnel, including bipolar disorder, obsessive-compulsive disorder, and phobias [4, 21]. However, our K10 cut-offs may still identify those at risk for other disorders that cause similar levels of distress as those assessed in the present survey. Second, our survey did not include other measures of generalized distress, against which convergent validity evidence could be optimally assessed. Third, imposing any single cut-off (no matter how rigorously established) for a continuous construct like psychological distress measured by the K10 will always result in loss of information and misclassification. We did indeed find a meaningful amount of misclassification (false negatives plus false positives ~ 6.6%). Finally, the predictive value of the K10 in clinical screening programs (in which barriers to disclosure may exist, especially in military personnel) [46] may differ from that demonstrated in the present analysis.

Strengths of this study include validation of the K10 against a reliable outcome (disorders assessed by the CIDI), the generous sample size, and the high response rate. Another strength relates on the identification of cutoffs for both screening and for estimation of the prevalence of mood, anxiety, and post-traumatic disorders.

### Implications for research and clinical practice

Taken as a whole, these findings provide satisfactory convergent validity evidence of the K10 in the Canadian military population. Specifically, the CFA findings and internal consistency provide reassurance as to internal structure as a source of validity evidence (that is, that the measure is unidimensional as intended). The association of the K10 with outcomes known to be associated with distress and mental disorders provides further evidence of construct validity. Its negative association with positive mental health also provides convergent evidence of validity. Finally, its association with the presence and recency of mood, anxiety, and post-traumatic disorders provide further support that the K10 was designed to accurately predict the presence of mental disorders, and it did so in our population.

Those wishing to use the scale as a dependent or independent variable in future research or as a screening tool can thus do so with knowledge of its psychometric properties and the extent to which these provide evidence of its validity as a tool for the estimation of the prevalence of common mental disorders (and hence, as a potential tool for screening) in the Canadian military population. However, those using the scale in any population should be aware of the somewhat unusual objectives in its development: to efficiently and pragmatically assess the prevalence of a range of disorders constituting “serious mental illness” in a population.

Our presentation of the full range of ROC indices will allow researchers and clinicians to select a cut-off (or cut-offs) that meet their needs. Topics of future research with the K10 in military populations might include exploration of the reasons behind the observed misclassification. False positive cases may represent those who mental disorders other than those assessed on the survey or they may represent those with elevated distress in the absence of a disorder. False negative cases may represent those with either mild or sub-threshold forms (limited symptom burden, limited functional impact) of the underlying disorder. Exploration of different scoring approaches to the K10 items (such as those identified through item response theory analysis) might ultimately lead to greater predictive value and hence greater research and clinical utility [7].

## Conclusion

The present study suggests that K10 scale has psychometric properties that favour its use as a measure of generalized psychological distress in Canadian military personnel, both for the purposes of screening and estimation of the prevalence of past-month mood, anxiety, and post-traumatic disorders.
